# Cast-OFF Trial: One Versus 4 to 5 Weeks of Plaster Cast Immobilization for Nonreduced Distal Radius Fractures: A Randomized Clinical Feasibility Trial

**DOI:** 10.1177/15589447211044775

**Published:** 2021-09-27

**Authors:** Emily Z. Boersma, Edo J. Hekma, Nicole Kraaijvanger, Roland M. H. G. Mollen, Maria W. G. Nijhuis-van der Sanden, Michael J. R. Edwards

**Affiliations:** 1 Radboud University Medical Center, Nijmegen, The Netherlands; 2 Rijnstate Hospital, Arnhem, The Netherlands; 3 Ziekenhuis Gelderse Vallei, Ede, The Netherlands

**Keywords:** distal radius, fracture/dislocation, diagnosis, nonoperative treatment, wrist, nonreduced, pain management, specialty

## Abstract

**Background:**

Distal radius fracture is a common fracture of which the incidence appears to be increasing worldwide. This pilot study investigated whether 1 week of plaster cast is feasible for nonreduced (stable fractures including nondisplaced and displaced fractures) distal radius fractures.

**Methods:**

The study was a multicenter randomized clinical feasibility trial including patients from regional acute care providers. Patients with a nonreduced distal radius fracture were included in the study. Nonreduced fractures meant intra-articular or extra-articular fractures and including nondisplaced and minimal displaced fractures (dorsal angulation less than 5°-10°, maximum radial shortening of 2 mm, and maximum radial shift of 2 mm) not needing a reduction. Forty Patients were included and randomized. After 1 week of plaster cast, patients were randomized to 1 of the 2 treatment groups: plaster cast removed (intervention group) versus 4 to 5 weeks of plaster cast (control group).

**Results:**

The analysis shows no significant differences between the 2 groups in having less pain, better function after 6 weeks, and better overall patient satisfaction. No difference was shown in secondary displacement between the 2 groups (control 1 vs intervention 0)

**Conclusion:**

One week of plaster cast treatment for nonreduced distal radius fracture is feasible, preferred by patients, with at least the same functional outcome and pain scores.

**Level of Evidence:**

According to the Oxford 2011 level of evidence, the level of evidence of this study is 2.

## Introduction

Distal radius fracture (DRF) is a common fracture of which the incidence appears to increase worldwide.^1,2^ Literature has mainly focused on treatment options for unstable DRF for which several treatment modalities have been advocated.^3,4^ To date, there are only a few studies that have investigated the duration of immobilization for nonoperatively treated, stable DRFs. A systematic review from 2018 studied the duration of immobilization for these fractures. The study showed that an immobilization period of 3 weeks or less is equally effective compared with longer immobilization and might even be associated with better functional outcome.^5^

In the Dutch DRF guideline (2010), the treatment advice for a nonreduced DRF is plaster cast or brace immobilization varying between 1 and 3 weeks.^3^ Despite this advice in the guideline and evidence from the literature,^5^ the usual length of plaster cast treatment for a stable or nonreduced DRF varies between 4 and 6 weeks. Recent studies have shown that a long period of immobilization can lead to more risk of post-traumatic pain including complex regional pain syndrome (CRPS).^6-8^

Based on the literature and keeping the discomfort plaster cast can give to patients in mind, this study will give a first answer on whether plaster cast for 1 week is feasible for nonreduced DRF. This study will investigate whether 1 week of plaster cast immobilization is safe with at least the same complication rate, and will lead to better or the same functional results and pain scores as 4 to 5 weeks of plaster cast immobilization. In addition, the results of this study will be used to design a larger national study.

## Methods

### Study Design

The study was a multicenter randomized clinical feasibility trial and was performed from July 2017 until December 2019. Adult patients with a nonreduced DRF were included in the study. After 1 week of plaster cast immobilization, patients were randomized to 1 of the 2 treatment groups: 4 to 5 weeks of immobilization (control group) versus plaster cast removed (intervention group) (Supplemental Figure S1). Outcomes were measured at baseline (1 week after trauma), week 4 to 5, week 6, and at 3, 6, and 12 months.

Patients were treated, included, and randomized in the Radboud university medical center and two regional hospitals Gelderse Vallei and Rijnstate in the Netherlands. These hospitals collaborate as regional acute care providers. We included and randomized 40 patients.

### Participants

Patients with an acute nonreduced DRF who were diagnosed at the emergency department of the 3 participating hospitals were eligible for inclusion in the study. The inclusion criteria were women/men between the age of 18 and 75 years with an isolated, nonreduced DRF. Nonreduced fractures meant intra-articular or extra-articular fractures and including nondisplaced and minimal displaced fractures (dorsal angulation less than 5°-10°, maximum radial shortening of 2 mm, and maximum radial shift of 2 mm) not needing a reduction. Patients were to have a good understanding of the Dutch language. All patients who did not receive a reduction, decided by their treating emergency physician, were eligible for inclusion.

### Procedure

In the emergency department, the treating physician explained the study and gave the patient the information letter and informed consent form. All patients were treated with a plaster splint for 1 week in slightly volar and ulnar deviation. After 1 week, all patients were seen at the hospital outpatient clinic (plaster room). Patients who were eligible for the study and gave written informed consent were included and randomized for 1 of the 2 groups: change to circular plaster cast (control group) or splint removal (intervention group). The randomization was performed by using a randomization program from the Castor database which allowed for a blind allocation of participants. All patients who participated in the study were asked baseline questions about demographic variables, pain, and use of pain medication.

For the intervention group, the splint was removed after 1 week at the outpatient clinic. Patients were offered to use an elastic tubular support bandage. After 4 to 5 weeks after injury, these patients were seen a second time at the outpatient clinic. During this visit, an examination of the wrist (range of motion and neurovascular status of the hand and wrist were examined) was performed. Additional questions were asked about the functioning of their arm, pain, use of pain medication, and return to work for the last 3 to 4 weeks.

For the control group, usual care was performed: a visit to the outpatient clinic after 1 week for a plaster cast change to a circular cast following the Dutch guidelines. After 4 to 5 weeks after injury, these patients were seen for the second time at the outpatient clinic where the plaster cast was removed. The same examinations and questions were asked as for the intervention group.

For both groups, patients were given a home exercise program after removal of the plaster cast, with additional verbal instructions about the importance of using their arm and performing the exercises at home.

Follow-up for all participants took place with questionnaires at 6 weeks and 3, 6, and 12 months after injury. Questionnaires were sent to the patients via e-mail, and they were interviewed over telephone for extra follow-up questions if necessary. The study design is shown in Supplemental Figure S1.

### Outcome Measures

**Figure 1.**
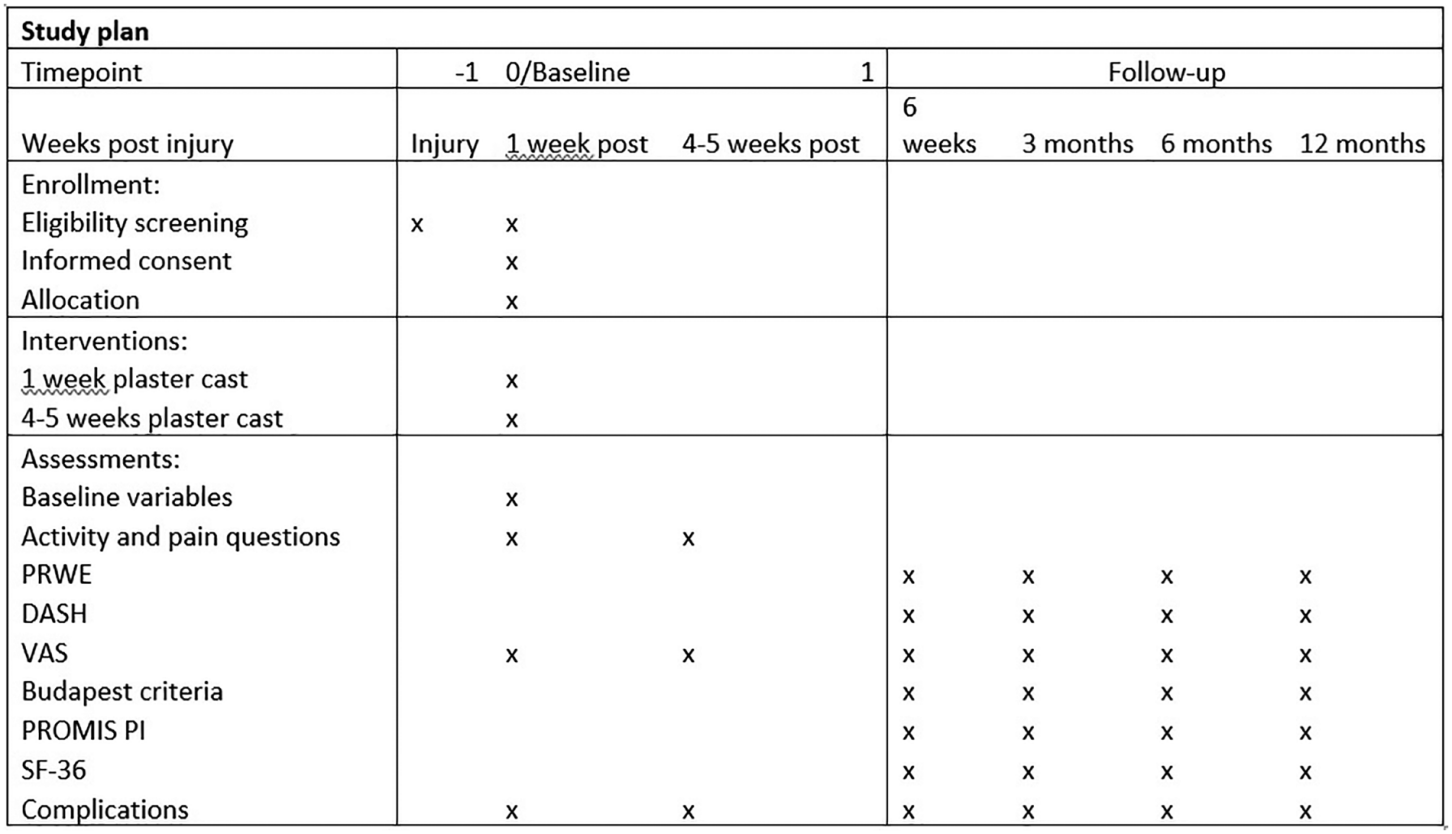
Cast-OFF trial follow-up schedule. Weeks 1 and 4 are follow-up appointments at the outpatient clinic. From week 6, the follow-up took place via questionnaires. *Note.* PRWE = Patient-Rated Wrist Evaluation; DASH = Disabilities of the Arm, Shoulder, and Hand; VAS = Visual Analog Scale; PROMIS PI = Patient-Reported Outcomes Measurement Information System Pain Interference; SF-36 = Short Form-36.

Patients were asked several questions during the outpatient clinic visits at 1 week and 4 to 5 weeks after injury, see Supplemental Material S2 and Figure 1. The follow-up period consisted of an outpatient clinic visit after 4 weeks and questionnaires sent at 6 weeks and 3, 6, and 12 months after injury. The questionnaires were sent via e-mail (Figure 1). The questionnaires included the Patient-Rated Wrist Evaluation (PRWE) score, Disabilities of the Arm, Shoulder, and Hand (DASH) Questionnaire, Patient-Reported Outcomes Measurement Information System (PROMIS) Pain Interference and Visual Analog Scale (VAS) score, and the Short Form-36 (SF-36).^6,9-19^ Complications were scored using the complication checklist for DRF from McKay et al,^20^ and questions about pain medication, work absence, patient satisfaction, and the experiences with the study participation were asked. The Budapest diagnostic criteria were used for scoring CRPS.^21^

After 1 year, the patients were asked whether they were treated by a physiotherapist for their wrist over the past year. See Supplemental Material S3 for information on the questionnaires used.

### Process Evaluation

As this was a feasibility study, a process evaluation was added. The evaluation focused on: (1) Willingness of participant. Reasons for not willing to participate, if voluntarily provided, were gathered and analyzed. In addition, participants were asked to give feedback on the study design and give their reaction to the intervention; and (2) the study design was evaluated afterwards. This included evaluation of the inclusion process, the intervention, and the follow-up with questionnaires.

### Sample Size Calculation

This study was a feasibility study and therefore did not include a power analysis. Results will be used for a sample size calculation for a larger trial.

### Statistical Analysis

An intention-to-treat (ITT) and per-protocol analysis was performed to compare the outcomes between the control and intervention group. For the per protocol analysis, we compared the intervention and control group, not based on the randomization but based on the treatment they received. Descriptive analysis with median, range, and percentage was used to describe demographic variables.

All reported *P* values are 2-sided and are considered significant at *P* < .05.

For missing data from patients who fulfilled the study, we analyzed the available data.

The results from the PRWE, DASH, pain indicated on a VAS and with the PROMIS Pain Interference score, and Quality of life (SF-36) were analyzed with a bivariate analysis. The association between Patient-Rated Outcome (PRO) scores and treatment group was investigated in multilevel linear mixed model with an unstructured parameterization for longitudinal covariance. Age, sex, VAS pain score, and fracture classification were used as fixed factors with time as the random factor. We used a random intercept and slope model. To investigate whether rate of change in PRO score over time was different between the intervention and control group, we studied the interaction between treatment (control vs intervention) group and time. We used the per-protocol analysis for the multilevel linear mixed analysis. The difference in complication rate was determined using a Fisher exact test.

## Results

### Patient Characteristics

**Figure 2.**
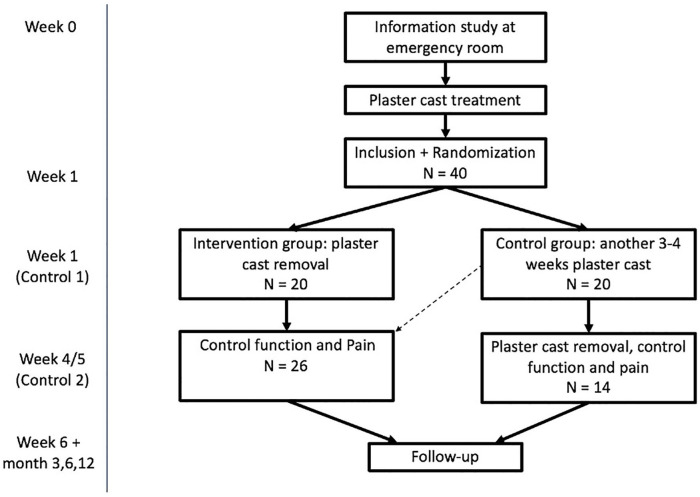
Cast-OFF study design, including crossover patients.

**Figure 3.**
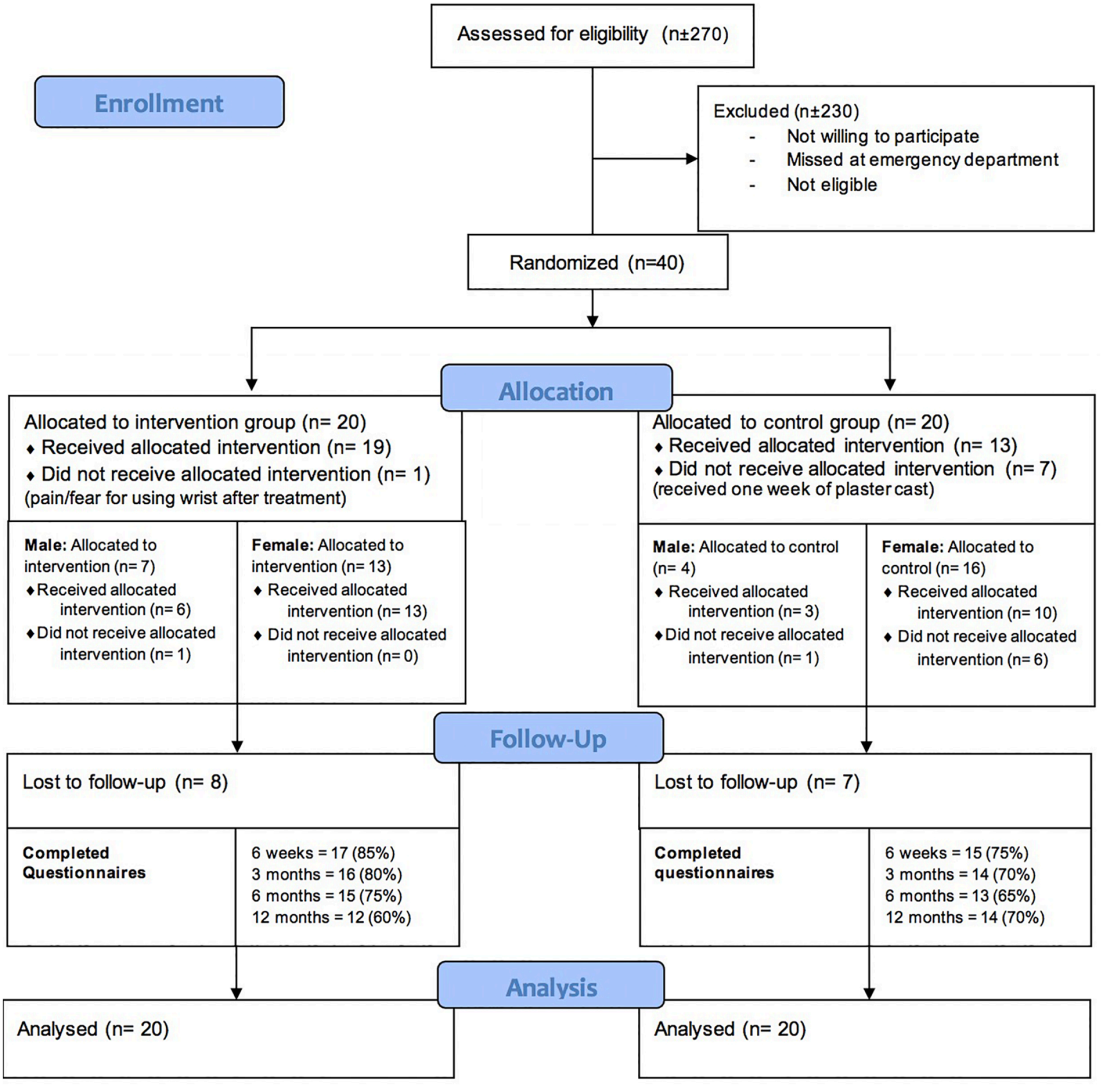
Cast-OFF trial CONSORT flow diagram.

A total of 40 patients were included in this randomized controlled trial feasibility study. Twenty patients were randomized in the intervention group and 20 in the control group; eventually, 14 patients were treated following the control group protocol and 26 patients following the intervention group protocol due to crossover, see Figures 2 and 3. See Table 1 for patient characteristics.

**Table 1.** Patient Characteristics.

Variables	Intention to treat		
	Control	Intervention	*P* value
	N = 20	N = 20	
Age, y	59 ± 9.9	48 ± 16.0	.**012**
Sex (female)	16 (80)	13 (65)	.48
Dominant hand^a^	18 (90)	18 (90)	1.0
Fracture history^b^	0 (0)	1 (5)	1.0
Smoking	0 (0)	3 (15)	.23
VAS score	2.6 ± 1.8	2.0 ± 1.4	.24
Pain medication	8 (40)	7 (35)	1.0
Vitamin C	7 (35)	6 (30)	1.0
AO classification			
23A	4 (20)	8 (40)	.49
23B	7 (35)	5 (25)	
23C	9 (45)	7 (35)	
Variables	Per protocol		
	N = 14	N = 26	*P* value
Age, y	56.5 ± 9.6	52.3 ± 16.2	.38
Sex (female)	10 (71)	19 (73.1)	1.0
Limb dominance^a^	13 (92.9)	23 (88.5)	1.0
Fracture history^b^	1 (7.14)	0 (0)	.35
Smoking	0 (0)	3 (11.5)	.54
VAS score	3.1 ± 1.9	1.9 ± 1.3	.**024**
Pain medication	6 (42.9)	9 (34.6)	.74
Vitamin C	6 (42.9)	7 (26.9)	.48
AO classification			
23A	2 (14)	10 (38.4)	.32
23B	5 (35)	7 (26.9)	
23C	7 (50)	9 (34.6)	

*Note.* Continuous variables as mean ± standard deviation; discrete variables as number (percentage). VAS = Visual Analog Scale.

a Fracture at dominant limb.

b Fracture in the past at the same side.

### Four Weeks After Injury

Using the ITT analysis, no significant difference was found between the 2 groups for general health, VAS score after 4 weeks, disability, activity, limitations due to pain or fear, use of pain medication, and use of vitamin C, see Table 2. Using the per-protocol analyses (patients analyzed in the groups they were treated), there were also no significant differences between the 2 groups, see Table 2.

**Table 2.** Four Weeks After Injury (DRF).

Variables	Intention to treat			Per protocol		
	Control	Intervention	*P* value	Control	Intervention	*P* value
	N = 20 (50%)	N = 20 (50%)		N = 14 (35%)	N = 26 (65%)	
General health						
Poor	0 (0)	0 (0)	.26	0 (0)	0 (0)	.15
Fair	1 (5)	2 (10)		1 (7.14)	2 (7.7)	
Good	14 (70)	8 (40)		10 (71.4)	12 (46.2)	
Very good	3 (15)	8 (40)		1 (7.14)	10 (38.5)	
Excellent	2 (10)	2 (10)		2 (14.3)	2 (7.7)	
VAS after 4 weeks	2.2 ± 1.9	2.0 ± 1.7	.73	1.9 ± 2.0	2.2 ± 1.7	.62
Disability						
Not at all	3 (15)	4 (20)	.19	1 (7.14)	6 (23.1)	.18
Slightly	14 (70)	8 (40)		11 (78.6)	11 (42.3)	
Moderately	3 (15)	7 (35)		2 (14.3)	8 (30.3)	
Quite a bit	0 (0)	1 (5)		0 (0)	1 (3.9)	
Extremely	0 (0)	0 (0)		0 (0)	0 (0)	
Activity						
Not at all	6 (30)	5 (25)	.55	4 (28.6)	7 (26.9)	.29
Slightly	10 (50)	13 (65)		6 (42.9)	17 (65.4)	
Moderately	1 (5)	2 (10)		2 (14.3)	1 (3.9)	
Quite a bit	2 (10)	0 (0)		1 (7.14)	1 (3.9)	
Extremely	1 (5)	0 (0)		1 (7.14)	0 (0)	
Limitations due to pain						
No	14 (70)	9 (45)	.20	9 (64.3)	14 (53.9)	.74
Yes	6 (30)	11 (55)		5 (35.7)	12 (46.1)	
Limitations due to fear						
No	17 (85)	14 (70)	.45	11 (78.6)	20 (76.9)	1
Yes	3 (15)	6 (30)		3 (21.4)	6 (23.1)	
Pain medication						
No	15 (75)	18 (90)	.41	10 (71.4)	23 (88.5)	.21
Yes	5 (25)	2 (10)		4 (28.6)	3 (11.5)	
Vitamin C						
No	13 (65)	14 (70)	1.00	8 (57.1)	19 (73.1)	.48
Yes	7 (35)	6 (30)		6 (42.9)	7 (26.9)	

*Note.* Bold value indicates significant difference; continuous variables as mean ± standard deviation; discrete variables as number (percentage). DRF = distal radius fracture; VAS = Visual Analog Scale.

### Patient-Reported Outcome

After 6 weeks, using the ITT analysis, the intervention group scored significantly lower on the DASH score and PROMIS PI (DASH control 28.8 vs intervention 15.6, *P* = .05 and PROMIS PI control 56.7 vs intervention 51.6, *P* = .04). After 3 and 12 months, the intervention group scored significantly lower on the PROMIS PI (3 months: 49.9 vs 44.2, *P* = .03; 12 months: 47.0 vs 41.5, *P* = .03). See Table 3 for the results of the PRWE, DASH, and PROMIS PI scores after 6 weeks and 3, 6, and 12 months. Using the per-protocol analyses, there was no significant difference between the control group and the intervention group for the DASH, PRWE, and PROMIS PI score. However, after 6 weeks, an advantage for the intervention group was shown in lower scores for the DASH, PRWE, and PROMIS PI, see Table 3.

**Table 3.** Patient Outcome Scores.

Variables	Intention to treat			Per protocol		
	Control	Intervention	*P* value	Control	Intervention	*P* value
	N = 20 (50%)	N = 20 (50%)		N = 14 (35%)	N = 26 (65%)	
6 weeks						
DASH	28.8 ± 16.6	15.6 ± 15.5	.051	26.1 ± 16.5	18.3 ± 17.0	.286
DASH work	30.8 ± 23.4	26.8 ± 27.7		34.4 ± 22.5	25.9 ± 26.9	
DASH hobbies	35.7 ± 32.0	25.4 ± 23.7		27.5 ± 31.6	31.3 ± 26.5	
PRWE	33.1 ± 18.5	24.5 ± 22.1	.253	32.1 ± 16.9	26.4 ± 2.6	.471
PRWE Pain	17.1 ± 10.6	14.1 ± 12.0		17.4 ± 9.9	14.5 ± 12.1	
PRWE Function	15.6 ± 8.4	10.6 ± 10.4		14.7 ± 7.7	11.8 ± 10.8	
PROMIS PI	56.7 ± 6.8	51.6 ± 6.4	.**042**	55.5 ± 7.5	53.3 ± 6.8	.404
3 months						
DASH	10.6 ± 6.7	9.3 ± 10.6	.760	10.5 ± 7.3	9.4 ± 10.2	.794
DASH work	11.4 ± 9.2	11.3 ± 18.7		7.1 ± 9.1	12.8 ± 16.9	
DASH hobbies	19.3 ± 33.7	15.8 ± 17.0		15.6 ± 23.9	18.1 ± 26.0	
PRWE	15.8 ± 15.3	10.2 ± 11.4	.289	16.3 ± 16.2	10.9 ± 11.7	.333
PRWE Pain	7.2 ± 7.6	6.3 ± 6.7		7.4 ± 8.0	6.4 ± 6.7	
PRWE Function	8.1 ± 8.7	3.9 ± 5.3		8.3 ± 9.5	4.5 ± 5.6	
PROMIS PI	49.9 ± 7.0	44.2 ± 6.7	.**029**	46.7 ± 6.6	46.9 ± 7.8	.924
6 months						
DASH	6.8 ± 5.7	5.7 ± 8.6	.730	6.0 ± 6.2	6.2 ± 8.1	.953
DASH work	3.1 ± 5.0	4.6 ± 12.6		3.5 ± 5.5	4.2 ± 11.5	
DASH hobbies	5.7 ± 7.8	4.6 ± 8.3		4.2 ± 7.0	5.6 ± 8.5	
PRWE	10.9 ± 12.7	7.2 ± 12.0	.436	10.7 ± 13.6	8.1 ± 11.8	.593
PRWE Pain	7.6 ± 9.8	5.2 ± 9.1		7.5 ± 11.0	5.8 ± 8.7	
PRWE Function	3.2 ± 3.8	2.4 ± 3.3		3.2 ± 3.6	2.6 ± 3.5	
PROMIS PI	45.8 ± 5.7	44.4 ± 5.6	.488	44.6 ± 5.1	45.3 ± 5.9	.727
12 months						
DASH	6.6 ± 10.3	1.6 ± 2.5	.115	3.5 ± 4.1	4.6 ± 9.5	.729
DASH work	8.7 ± 21.3	2.1 ± 7.2		4.2 ± 8.8	6.3 ± 19.4	
DASH hobbies	3.6 ± 7.8	3.1 ± 7.7		3.1 ± 8.8	3.5 ± 7.2	
PRWE	2.3 ± 3.1	2.9 ± 7.6	.818	2.1 ± 3.3	2.9 ± 6.6	.722
PRWE Pain	1.2 ± 2.3	1.6 ± 4.2		1.6 ± 2.7	1.3 ± 3.6	
PRWE Function	1.1 ± 1.9	1.3 ± 3.4		0.5 ± 0.9	1.6 ± 3.3	
PROMIS PI	47.0 ± 7.8	41.5 ± 2.7	.**028**	46.3 ± 7.3	43.3 ± 5.9	.262

*Note.* Bold value indicates significant difference; continuous variables as mean ± standard deviation; discrete variables as number (percentage). DASH = Disabilities of the Arm, Shoulder, and Hand; PRWE = Patient-Rated Wrist Evaluation; PROMIS PI = Patient-Reported Outcomes Measurement Information System Pain Interference.

### Multilevel Linear Mixed Analysis

In multilevel linear mixed model analysis adjusted for age, sex, VAS score at baseline, fracture classification, and time of data collection, there were no differences in DASH score, PRWE score, and PROMIS PI score between patients who were randomized in the control group or the intervention group (respectively, DASH: coeff = −1.97; 95% confidence interval [CI], −9.49 to 5.55, *P* = .61; PRWE: coeff = −4.91; 95% CI, −11.23 to 1.1, *P* = .13; PROMIS PI: coeff = −3.52; 95% CI, −7.61 to 0.57, *P* = .09). There was no statistically significant interaction between treatment and time for any of the PRO scores (*P* = .76 for DASH, *P* = .45 for PRWE, and *P* = .55 for PROMIS PI). However, in general, all PRO scores decreased over time, with the highest scores after 6 weeks. There were 28 individuals with at least 2 measurements of DASH score. This number was 30 and 31 for PRWE score and PROMIS Pain Interference score, respectively.

### Short Form-36

After 6 weeks, the intervention group scored better on the physical function, general health, and health change sections (control vs intervention: physical function, 68.7 vs 86.8, *P* = .02; general health, 60.3 vs 83.5, *P* < .01; health change, 41.7 vs 54.4, *P* < .01). After 3, 6, and 12 months, the intervention group scored better for the section pain (control vs intervention: pain 3 months, 69.4 vs 85.9, *P* = .04; pain 6 months, 75.6 vs 90.2, *P* = .04; pain 12 months, 75.7 vs 93.8, *P* = .02) see Supplemental Material S4. The per protocol showed no significant difference between the groups except for emotional problems after 12 months (control vs intervention: 80.0 vs 100.0, *P* = .03) see Supplemental Material S4.

### Complications

One secondary displacement was seen in the control group (treated with 4- to 5-week immobilization, secondary displacement did not need correction). In the control group, 2 patients were scored for bone and joint complications (ulnar-sided wrist pain [USWP] and distal radial ulnar joint pain) and 1 with tendon complaints (diagnosed with tendovaginitis). In the intervention group, 1 patient was diagnosed with USWP, see Table 4.

**Table 4.** Complications.

Variables	Control	Intervention
	N = 14 (35%)	N = 26 (65%)
Secondary dislocation	1 (7.1)	0 (0)
Nerve	0 (0)	0 (0)
Bone/Joint	2 (14.3)	1 (3.9)
Tendon	1 (7.1)	0 (0)
Other	0 (0)	0 (0)
CRPS	0 (0)	0 (0)
CRPS subjective 6 wk	6	7
CRPS subjective 3 mo	1	3
CRPS subjective 6 mo	0	2
CRPS subjective 12 mo	0	1

*Note.* Discrete variables as number (percentage). CRPS = complex regional pain syndrome.

After 6 weeks, 13 patients scored positive for post-traumatic pain, the subjective criteria of the Budapest criteria for CRPS (control, n = 6; intervention, n = 7), see Table 4. No patients were diagnosed with CRPS over 1-year follow-up.

### Process Evaluation

Crossover from 1 treatment group to the other took place in 8 patients; 7 patients went from the control group to the intervention group due to preference of the patients (n = 4) or by mistake by the plaster technician (n = 3). One patient crossed from the intervention to the control group due to pain complaints, see Figure 3. The mean age for the patient who switched from the control group to the intervention group was 65.1. The mean VAS score at baseline was 1.6. See Supplemental Material S5.

The follow-up with questionnaires showed a completion rate of 63% after 12 months. Especially, the DASH score was not completed. Patients found the questionnaires too long.

Patients were asked when they returned to work after injury. The mean was 2.8 weeks, and there was no difference between the 2 groups (both mean: 2.8 weeks).

## Discussion

Distal radius fracture is a common fracture of which the incidence appears to be increasing worldwide.^1,2^ Little is known about the optimal treatment for nonreduced DRF. This study investigated whether 1 week of plaster cast treatment is feasible for nonreduced DRF. One week of plaster cast treatment seems safe, preferred by patients, and has at least the same functional outcome and pain scores compared with 4 to 5 weeks of plaster cast immobilization.

Some limitations for this study are acknowledged. First, several patients changed from treatment group. Owing to the crossover, a good ITT analysis was not possible. However, as this is a feasibility study, it showed that patients prefer the intervention group. Second, 63% of the patients completed the entire follow-up. This could have biased the results. Especially, the DASH questionnaire seemed too long to complete, as was mentioned in the feedback from the patients. Third, patient characteristics, especially age, were significantly different between the 2 groups when using the ITT analysis. Owing to the small group, this could be explained by the 5% chance of difference between the randomization groups. In addition, the number of crossover patients could have influenced the diversity of the groups. For future studies, this could be prevented by using stratification during randomization. Finally, the randomization was impossible to be blinded for physician and patients. However, the randomization was blinded for the researcher.

Although DRF is 1 of the most common fractures seen in the emergency department, there is a large variation in the management of DRF. A study conducted at the emergency department found that especially young doctors had little awareness of anatomical parameters, criteria for reduction, and treatment options.^22^ Another study found that educating medical staff regarding fracture classifications, fracture reduction, and cast application significantly reduced the treatment variation between the medical staff.^23^ These studies show that more evidence on the treatment of nonreduced DRF is needed, which will eventually lead to less variation. Especially for nonreduced DRF, only a few studies have looked at the duration for plaster cast treatment and the majority of these studies were conducted at least 2 decades ago.^5^

During the last couple of years, a tendency is shown in shorter immobilization periods for nonreduced including nondisplaced and minimal displaced DRF. However, the usual practice is often still an immobilization period for 4 to 5 weeks. A systematic review on duration of cast immobilization for DRF from 2018 showed that an immobilization period of 3 weeks or less is equally effective compared with longer immobilization period and might be associated with better functional outcome.^5^ These findings are consistent with the results from this study, which showed that 1 week of plaster cast immobilization for nonreduced DRF is feasible with at least the same functional outcome and pain scores as the control group (immobilization for 4-5 weeks). One week of immobilization for nondisplaced DRF seems feasible, but more up-to-date research is needed to eventually proof that shorter immobilization period leads to better (functional) outcome.

This study found no difference in the complication rate between the 2 groups and found 1 secondary displacement in the control group, which was treated nonoperatively with plaster cast immobilization for a total of 5 weeks. This is consistent with the literature. The studies by Davis and Buchanan,^24^ Jensen,^25^ and Stoffelen and Broos^26^ also found no increase in secondary displacement with 1 week of plaster cast immobilization for nondisplaced and minimally displaced fractures. However, the study of Christersson and colleagues^27^ shows that moderately displaced DRF, treated with closed reduction and plaster cast immobilization for 10 days, resulted in an increased number of secondary displacements. This is supported by the study of Jung and colleagues who described that greater displacement on the initial radiographs gives a higher possibility of early redisplacement (within 2 weeks after injury). In addition, age seems to be associated with redisplacement (after 2 weeks).^28^ It could be argued that selecting only nonreduced DRF is safe and will not lead to secondary displacement and therefore is the right group for 1-week immobilization. However, further studies should investigate the risk of secondary displacement for patients older than 65 years when treated with 1 week of plaster cast immobilization.

The intervention group seemed to have a better patient satisfaction. Eventually, more patients were treated with 1 week of plaster cast. We did not see a difference in return to work, compared with the control group. Both groups had a mean of 2.8 weeks before they returned to work. However, scoring return to work is very difficult due to the large difference in occupational demand. Very little is known about the influence of DRF on work loss. The study of MacDermid and colleagues^29^ found that loss of work was highly variable after DRF and could only find that high self-reported pain/disability and occupational demand at baseline were at risk for prolonged work loss. During a future study, work loss should be investigated and occupational demand should be taken into account.

## Conclusion

Based on this feasibility study, 1 week of plaster cast treatment for nonreduced DRF is feasible, preferred by patients, with at least the same functional and pain scores as treatment with 4 to 5 weeks of plaster cast immobilization. There were no differences in complications and secondary displacements. Owing to these positive results, a randomized multicenter stepped wedge design will be conducted, and hopefully, 1 week of plaster cast treatment can be implemented in daily practice.

## Supplemental Material

sj-jpg-1-han-10.1177_15589447211044775 – Supplemental material for Cast-OFF Trial: One Versus 4 to 5 Weeks of Plaster Cast Immobilization for Nonreduced Distal Radius Fractures: A Randomized Clinical Feasibility Trial Click here for additional data file. Supplemental material, sj-jpg-1-han-10.1177_15589447211044775 for Cast-OFF Trial: One Versus 4 to 5 Weeks of Plaster Cast Immobilization for Nonreduced Distal Radius Fractures: A Randomized Clinical Feasibility Trial by Emily Z. Boersma, Edo J. Hekma, Nicole Kraaijvanger, Roland M. H. G. Mollen, Maria W. G. Nijhuis-van der Sanden and Michael J. R. Edwards in HAND

sj-pdf-1-han-10.1177_15589447211044775 – Supplemental material for Cast-OFF Trial: One Versus 4 to 5 Weeks of Plaster Cast Immobilization for Nonreduced Distal Radius Fractures: A Randomized Clinical Feasibility Trial Click here for additional data file. Supplemental material, sj-pdf-1-han-10.1177_15589447211044775 for Cast-OFF Trial: One Versus 4 to 5 Weeks of Plaster Cast Immobilization for Nonreduced Distal Radius Fractures: A Randomized Clinical Feasibility Trial by Emily Z. Boersma, Edo J. Hekma, Nicole Kraaijvanger, Roland M. H. G. Mollen, Maria W. G. Nijhuis-van der Sanden and Michael J. R. Edwards in HAND

sj-pdf-2-han-10.1177_15589447211044775 – Supplemental material for Cast-OFF Trial: One Versus 4 to 5 Weeks of Plaster Cast Immobilization for Nonreduced Distal Radius Fractures: A Randomized Clinical Feasibility Trial Click here for additional data file. Supplemental material, sj-pdf-2-han-10.1177_15589447211044775 for Cast-OFF Trial: One Versus 4 to 5 Weeks of Plaster Cast Immobilization for Nonreduced Distal Radius Fractures: A Randomized Clinical Feasibility Trial by Emily Z. Boersma, Edo J. Hekma, Nicole Kraaijvanger, Roland M. H. G. Mollen, Maria W. G. Nijhuis-van der Sanden and Michael J. R. Edwards in HAND

sj-pdf-3-han-10.1177_15589447211044775 – Supplemental material for Cast-OFF Trial: One Versus 4 to 5 Weeks of Plaster Cast Immobilization for Nonreduced Distal Radius Fractures: A Randomized Clinical Feasibility Trial Click here for additional data file. Supplemental material, sj-pdf-3-han-10.1177_15589447211044775 for Cast-OFF Trial: One Versus 4 to 5 Weeks of Plaster Cast Immobilization for Nonreduced Distal Radius Fractures: A Randomized Clinical Feasibility Trial by Emily Z. Boersma, Edo J. Hekma, Nicole Kraaijvanger, Roland M. H. G. Mollen, Maria W. G. Nijhuis-van der Sanden and Michael J. R. Edwards in HAND

sj-pdf-4-han-10.1177_15589447211044775 – Supplemental material for Cast-OFF Trial: One Versus 4 to 5 Weeks of Plaster Cast Immobilization for Nonreduced Distal Radius Fractures: A Randomized Clinical Feasibility Trial Click here for additional data file. Supplemental material, sj-pdf-4-han-10.1177_15589447211044775 for Cast-OFF Trial: One Versus 4 to 5 Weeks of Plaster Cast Immobilization for Nonreduced Distal Radius Fractures: A Randomized Clinical Feasibility Trial by Emily Z. Boersma, Edo J. Hekma, Nicole Kraaijvanger, Roland M. H. G. Mollen, Maria W. G. Nijhuis-van der Sanden and Michael J. R. Edwards in HAND
