# Supplementary figures and images for: Cast-OFF Trial: One Versus 4 to 5 Weeks of Plaster Cast Immobilization for Nonreduced Distal Radius Fractures: A Randomized Clinical Feasibility Trial
Source: Hand (N Y). 2021 Sep 27;17(1 Suppl):60S–69S. doi: 10.1177/15589447211044775 (PMC9793615; doi:10.1177/15589447211044775)

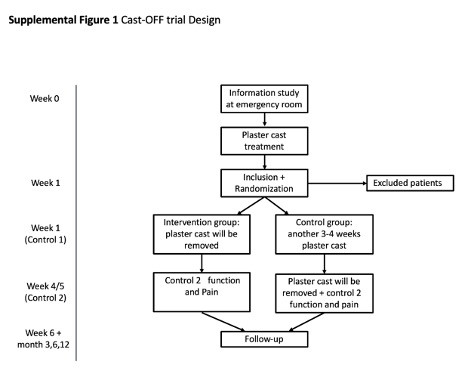

Supplement: sj-jpg-1-han-10.1177_15589447211044775 – Supplemental material for Cast-OFF Trial: One Versus 4 to 5 Weeks of Plaster Cast Immobilization for Nonreduced Distal Radius Fractures: A Randomized Clinical Feasibility Trial [file sj-jpg-1-han-10.1177_15589447211044775.jpg]
