# Supplementary material for: Cast-OFF Trial: One Versus 4 to 5 Weeks of Plaster Cast Immobilization for Nonreduced Distal Radius Fractures: A Randomized Clinical Feasibility Trial
Source: Hand (N Y). 2021 Sep 27;17(1 Suppl):60S–69S. doi: 10.1177/15589447211044775 (PMC9793615; doi:10.1177/15589447211044775)
Supplement: sj-pdf-1-han-10.1177_15589447211044775 – Supplemental material for Cast-OFF Trial: One Versus 4 to 5 Weeks of Plaster Cast Immobilization for Nonreduced Distal Radius Fractures: A Randomized Clinical Feasibility Trial [file sj-pdf-1-han-10.1177_15589447211044775.pdf]

Supplemental file 2 Questions asked during the outpatient clinic visit

<u>One-week visit at outpatient clinic</u>

1. Demographic questions consisting:

    1.1 Age

    1.2 Gender (male/female)

    1.3 Dominant arm (left/right)

    1.4 VAS score base line (10-point scale)

    1.5 Smoking (yes/no)

    1.6 Pain medication (yes/no), if yes what pain medication

    1.7 Vitamin C use (yes/no)

    1.8 Work/profession

    1.9 History of fracture of the arm (yes/no)

2. The AO classification system was used to classify the fractures (1), the treating physician classified the fractures and this was checked by EB [researcher].

<u>Four-Five weeks visit at outpatient clinic</u>

1. Questions for function, pain and return to activity last three weeks

    1.1 Function last week (5-point likert scale)

    1.2 VAS score after four to five weeks post injury

    (A scale from 0 to 10 where 0 is no pain at all and 10 is the worst pain imaginable)

    1.3 Hindrance of pain or plaster cast last week (5-point likert scale)

    1.4 Return to work or activities (5-point likert scale)

    1.5 Avoidance of activities due to pain (yes/no)

1.6 Avoidance of activities due to fear (yes/no)

2. Use of home exercise program (yes/no)

3. Use of pain medication (yes/no), if yes what pain medication

4. Use of the elastic tubular support bandage (yes/no)

5. Use of vitamin C (yes/no)

References

1. Medoff RJ. Essential radiographic evaluation for distal radius fractures. *Hand Clin*. 2005;21:279-288.
