# Supplementary material for: Cast-OFF Trial: One Versus 4 to 5 Weeks of Plaster Cast Immobilization for Nonreduced Distal Radius Fractures: A Randomized Clinical Feasibility Trial
Source: Hand (N Y). 2021 Sep 27;17(1 Suppl):60S–69S. doi: 10.1177/15589447211044775 (PMC9793615; doi:10.1177/15589447211044775)
Supplement: sj-pdf-2-han-10.1177_15589447211044775 – Supplemental material for Cast-OFF Trial: One Versus 4 to 5 Weeks of Plaster Cast Immobilization for Nonreduced Distal Radius Fractures: A Randomized Clinical Feasibility Trial [file sj-pdf-2-han-10.1177_15589447211044775.pdf]

Supplemental file 3 Measurements

Patient Rated Wrist Evaluation score (PRWE)

The Patient Rated Wrist Evaluation score (PRWE) is specifically designed for wrist and hand functioning. The PRWE is a reliable and valid measure of patient rated pain and disability for wrist conditions (1, 2). A validated Dutch version of the questionnaire is available (3). The PRWE is a measurement with a score from 0 to 100, where 0 is no wrist function problems or pain. The minimal clinical important difference for PRWE is a difference of 11 points (4).

Disability of the Arm, Hand and Shoulder Questionnaire (DASH)

The Disability of the Arm, Hand and Shoulder Questionnaire (DASH) questionnaire was used for upper extremity functioning (5-7). The DASH measurement consists of general function questions and a hobbies and work section and can be scored from 0 to 100 where 0 is no problems.

PROMIS Pain Interference and Visual Analogue Scale (VAS)

The PROMIS pain interference questionnaire was used for pain interference and diagnosing post traumatic pain (8, 9). The PROMIS Pain Interference consists of 8 questions. A difference of 3 points is assessed to be a relevant clinical difference (10). In addition to the PROMIS Pain Interference the visual analogue scale (VAS) was asked. A scale from 0 to 10 where 0 is no pain at all and 10 is the worst pain imaginable.

Short Form - 36 (SF-36)

The Short Form – 36 (SF-36) was used to measure the general quality of life. The RAND SF-36 was used and consists of different different sections: physical function, role of limitations due to physical health, role of limitations due to emotional problems, energy/fatigue, emotional well-being, social functioning, pain, general health. Every section can be scored from 0 to 100, where 100 is no problem/pain (11-13).

Complications

The complication checklist for DRF from McKay was used for scoring the complications after a DRF (14). Electronic patient records, questions by interview and questionnaires were used to complete the checklist. The Budapest diagnostic criteria was used to score CRPS, a complication which can occur after a DRF (15).

References

1. MacDermid JC, Turgeon T, Richards RS, et al. Patient rating of wrist pain and disability: a reliable and valid measurement tool. *J Orthop Trauma*. 1998;12:577-586.
2. Mehta SP, MacDermid JC, Richardson J, et al. A systematic review of the measurement properties of the patient-rated wrist evaluation. *J Orthop Sports Phys Ther*. 2015;45:289-298.
3. El Moumni M, Van Eck ME, Wendt KW, et al. Structural Validity of the Dutch Version of the Patient-Rated Wrist Evaluation (PRWE-NL) in Patients With Hand and Wrist Injuries. *Phys Ther*. 2016;96:908-916.
4. Walenkamp MM, de Muinck Keizer RJ, Goslings JC, et al. The Minimum Clinically Important Difference of the Patient-rated Wrist Evaluation Score for Patients With Distal Radius Fractures. *Clin Orthop Relat Res*. 2015;473:3235-3241.
5. Gummesson C, Ward MM, Atroshi I. The shortened disabilities of the arm, shoulder and hand questionnaire (QuickDASH): validity and reliability based on responses within the full-length DASH. *BMC Musculoskelet Disord*. 2006;7:44.
6. MacDermid JC, Richards RS, Donner A, et al. Responsiveness of the short form-36, disability of the arm, shoulder, and hand questionnaire, patient-rated wrist evaluation, and physical impairment measurements in evaluating recovery after a distal radius fracture. *J Hand Surg Am*. 2000;25:330-340.

7. MacDermid JC, Roth JH, Richards RS. Pain and disability reported in the year following a distal radius fracture: a cohort study. *BMC Musculoskelet Disord*. 2003;4:24.
8. Friesgaard KD, Gromov K, Knudsen LF, et al. Persistent pain is common 1 year after ankle and wrist fracture surgery: a register-based questionnaire study. *Br J Anaesth*. 2016;116:655-661.
9. Overbeek CL, Nota SP, Jayakumar P, et al. The PROMIS physical function correlates with the QuickDASH in patients with upper extremity illness. *Clin Orthop Relat Res*. 2015;473:311-317.
10. Chen CX, Kroenke K, Stump TE, et al. Estimating minimally important differences for the PROMIS pain interference scales: results from 3 randomized clinical trials. *Pain*. 2018;159:775-782.
11. Hays RD, Morales LS. The RAND-36 measure of health-related quality of life. *Ann Med*. 2001;33:350-357.
12. Hays RD, Sherbourne CD, Mazel RM. The RAND 36-Item Health Survey 1.0. *Health Econ*. 1993;2:217-227.
13. Saimanen I, Kuosmanen V, Rahkola D, et al. RAND-36-Item Health Survey: A Comprehensive Test for Long-term Outcome and Health Status Following Surgery. *Anticancer Res*. 2019;39:2927-2933.
14. McKay SD, MacDermid JC, Roth JH, et al. Assessment of complications of distal radius fractures and development of a complication checklist. *J Hand Surg Am*. 2001;26:916-922.
15. Harden RN, Bruehl S, Perez RS, et al. Validation of proposed diagnostic criteria (the "Budapest Criteria") for Complex Regional Pain Syndrome. *Pain*. 2010;150:268-274.
