# Supplementary material for: Cast-OFF Trial: One Versus 4 to 5 Weeks of Plaster Cast Immobilization for Nonreduced Distal Radius Fractures: A Randomized Clinical Feasibility Trial
Source: Hand (N Y). 2021 Sep 27;17(1 Suppl):60S–69S. doi: 10.1177/15589447211044775 (PMC9793615; doi:10.1177/15589447211044775)
Supplement: sj-pdf-3-han-10.1177_15589447211044775 – Supplemental material for Cast-OFF Trial: One Versus 4 to 5 Weeks of Plaster Cast Immobilization for Nonreduced Distal Radius Fractures: A Randomized Clinical Feasibility Trial [file sj-pdf-3-han-10.1177_15589447211044775.pdf]

Supplemental table. Short Form 36						
	Intention to treat			Per protocol		
	**Control**	**Intervention**		**Control**	**Intervention**	
Variables	**N = 20 (50%)**	**N = 20 (50%)**	**P-value**	**N = 14 (35%)**	**N = 26 (65%)**	**P-value**
6 weeks						
Physical function	68.7 ± 28.6	86.8 ± 10.9	**0.022**	68.7 ± 31.9	83.3 ± 14.5	0.082
Limitations physical health	43.3 ± 33.4	63.2 ± 37.6	0.126	54.5 ± 31.2	53.6 ± 39.8	0.944
Limitations emotional problems	51.0 ± 41.6	68.7 ± 36.3	0.208	60.6 ± 39.0	60.4 ± 40.3	0.988
Energy/fatigue	69.5 ± 17.4	77.9 ± 17.9	0.189	75.2 ± 12.0	73.3 ± 20.6	0.780
Emotional well-being	79.5 ± 15.6	86.6 ± 13.1	0.171	84.7 ± 12.6	82.5 ± 15.7	0.685
Social function	83.2 ± 18.1	86.2 ± 13.6	0.604	88.5 ± 19.7	82.9 ± 13.2	0.342
Pain	57.3 ± 22.7	71.2 ± 18.3	0.065	56.8 ± 22.0	68.8 ± 20.3	0.133
General health	60.3 ± 21.8	83.5 ± 10.7	**< 0.001**	64.1 ± 23.3	77.1 ± 17.4	0.084
Health change	41.7 ± 12.2	54.4 ± 9.8	**0.003**	43.2 ± 11.7	51.2 ± 12.4	0.088
3 months						
Physical function	75.4 ± 23.9	88.1 ± 15.3	0.094	76.7 ± 27.5	85.0 ± 16.3	0.318
Limitations physical health	75.0 ± 36.8	81.8 ± 33.9	0.612	80.6 ± 39.1	77.9 ± 33.7	0.853
Limitations emotional problems	92.3 ± 20.0	91.7 ± 14.8	0.930	96.3 ± 11.1	90.1 ± 19.0	0.369
Energy/fatigue	72.5 ± 19.5	70.6 ± 16.0	0.774	74.2 ± 18.0	70.3 ± 17.4	0.578
Emotional well-being	78.9 ± 13.4	80.8 ± 16.5	0.750	84.9 ± 8.2	77.7 ± 16.9	0.238
Social function	86.7 ± 15.7	95.3 ± 7.7	0.065	91.7 ± 8.8	91.4 ± 14.1	0.955
Pain	69.4 ± 23.1	85.9 ± 16.8	**0.035**	72.8 ± 23.0	81.1 ± 20.4	0.336
General health	63.8 ± 24.3	76.6 ± 16.1	0.103	63.9 ± 30.1	74.0 ± 15.0	0.233
Health change	44.3 ± 15.0	50.0 ± 9.1	0.212	41.5 ± 12.5	50.0 ± 11.5	0.089
6 months						
Physical function	78.1 ± 28.3	92.3 ± 15.1	0.103	82.1 ± 27.9	87.8 ± 20.3	0.537
Limitations physical health	80.8 ± 30.9	90.0 ± 26.4	0.401	87.5 ± 27.0	84.7 ± 29.9	0.810
Limitations emotional problems	87.2 ± 21.7	88.9 ± 27.2	0.856	93.3 ± 14.0	85.2 ± 28.5	0.407
Energy/fatigue	74.2 ± 28.6	77.7 ± 15.0	0.685	77.2 ± 23.9	75.6 ± 21.2	0.855
Emotional well-being	82.7 ± 17.5	84.8 ± 13.6	0.725	88.9 ± 7.4	81.3 ± 17.5	0.230
Social function	90.4 ± 22.3	96.7 ± 7.4	0.313	98.8 ± 4.0	91.0 ± 19.6	0.228
Pain	75.6 ± 19.3	90.2 ± 15.7	**0.036**	83.5 ± 13.5	83.3 ± 21.3	0.982
General health	70.7 ± 27.5	78.9 ± 18.4	0.364	71.5 ± 26.4	77.0 ± 21.6	0.562
Health change	48.1 ± 6.9	53.3 ± 8.8	0.094	47.5 ± 7.9	52.8 ± 8.1	0.107
12 months						
Physical function	80.0 ± 26.4	94.6 ± 7.5	0.077	78.0 ± 30.5	92.2 ± 9.8	0.095
Limitations physical health	82.5 ± 32.2	95.8 ± 9.7	0.181	78.0 ± 35.1	95.3 ± 13.6	0.086
Limitations emotional problems	85.7 ± 31.2	100.0 ± 0.0	0.128	80.0 ± 35.8	100.0 ± 0.0	**0.033**
Energy/fatigue	77.9 ± 15.7	74.6 ± 11.0	0.547	78.8 ± 17.1	74.7 ± 10.8	0.464
Emotional well-being	81.5 ± 12.7	84.0 ± 13.1	0.638	85.2 ± 12.5	81.1 ± 13.0	0.437
Social function	84.8 ± 22.0	96.9 ± 7.8	0.085	87.5 ± 23.6	92.2 ± 13.6	0.524
Pain	75.7 ± 24.0	93.8 ± 10.8	**0.023**	75.5 ± 26.9	89.4 ± 14.6	0.100
General health	66.9 ± 25.1	74.2 ± 22.7	0.459	65.5 ± 29.3	73.7 ± 19.8	0.412
Health change	48.2 ± 11.9	47.9 ± 7.2	0.940	45.0 ± 10.5	50.0 ± 9.1	0.212

Bold indicates significant difference; continuous variables as mean ± standard deviation; discrete variables as number (percentage).
