# Supplementary material for: Cast-OFF Trial: One Versus 4 to 5 Weeks of Plaster Cast Immobilization for Nonreduced Distal Radius Fractures: A Randomized Clinical Feasibility Trial
Source: Hand (N Y). 2021 Sep 27;17(1 Suppl):60S–69S. doi: 10.1177/15589447211044775 (PMC9793615; doi:10.1177/15589447211044775)
Supplement: sj-pdf-4-han-10.1177_15589447211044775 – Supplemental material for Cast-OFF Trial: One Versus 4 to 5 Weeks of Plaster Cast Immobilization for Nonreduced Distal Radius Fractures: A Randomized Clinical Feasibility Trial [file sj-pdf-4-han-10.1177_15589447211044775.pdf]

**Supplemental file 5. Patient characteristics for cross over patients**	
	**Patients**
Variables	**N = 7**
**Age**	65.1
**Gender (female)**	6
**Fracture history**	0 (0)
**Smoking**	0 (0)
**VAS score**	1.6
**Pain medication**	3
**Vitamin C**	1
**AO classification**	
23 A	2
23B	2
23C	3

Cross over patients, from control to intervention group; VAS = visual analogue scale.
